# Respiratory mycobiome and suggestion of inter-kingdom network during acute pulmonary exacerbation in cystic fibrosis

**DOI:** 10.1038/s41598-020-60015-4

**Published:** 2020-02-27

**Authors:** Perrine Soret, Louise-Eva Vandenborght, Florence Francis, Noémie Coron, Raphael Enaud, Marta Avalos, Thierry Schaeverbeke, Patrick Berger, Michael Fayon, Rodolphe Thiebaut, Laurence Delhaes, The Mucofong Investigation Group, Magali Chabe, Christophe Audebert, Isabelle Durand-Joly, Amale Boldron, Isabelle Pin, Odile Cognet, Herve Pelloux, Anne Prevotat, Benoit Wallaert, Nathalie Wizla, Caroline Thumerelle, Dominique Turck

**Affiliations:** 1 Univ. Bordeaux, Inserm, Bordeaux Population Health Research Center, UMR 1219, F-33000 Bordeaux, France; 2 INRIA SISTM Team, F-33405 Talence, France; 3 Univ. Bordeaux, Centre de Recherche Cardio-Thoracique de Bordeaux, U1045, F-33000 Bordeaux, France; 4 Genoscreen Society, 59000 Lille, France; 5 CHU Bordeaux, Department of Public Health, F-33000 Bordeaux, France; 6 CHU de Bordeaux, Univ. Bordeaux, FHU ACRONIM, F-33000 Bordeaux, France; 7 CHU de Bordeaux: Laboratoire de Parasitologie-Mycologie, Univ. Bordeaux, F-33000 Bordeaux, France; 8 CHU de Bordeaux, CRCM Pédiatrique, CIC, 1401 Bordeaux, France; 9 Present Address: University and CHU of Lille, F-59000 Lille, France; 10 Lille University, Lille, France; 11 Pegase consortium, Lille, France; 12 Dunkerque Hospital, Dunkerque, France; 13 Grenoble Hospital and University, Grenoble, France; 14 Lille Hospital and University, Lille, France

**Keywords:** Translational research, Infection

## Abstract

Lung infections play a critical role in cystic fibrosis (CF) pathogenesis. CF respiratory tract is now considered to be a polymicrobial niche and advances in high-throughput sequencing allowed to analyze its microbiota and mycobiota. However, no NGS studies until now have characterized both communities during CF pulmonary exacerbation (CFPE). Thirty-three sputa isolated from patients with and without CFPE were used for metagenomic high-throughput sequencing targeting 16S and ITS2 regions of bacterial and fungal rRNA. We built inter-kingdom network and adapted Phy-Lasso method to highlight correlations in compositional data. The decline in respiratory function was associated with a decrease in bacterial diversity. The inter-kingdom network revealed three main clusters organized around *Aspergillus*, *Candida*, and *Scedosporium* genera. Using Phy-Lasso method, we identified *Aspergillus* and *Malassezia* as relevantly associated with CFPE, and *Scedosporium* plus *Pseudomonas* with a decline in lung function. We corroborated *in vitro* the cross-domain interactions between *Aspergillus* and *Streptococcus* predicted by the correlation network. For the first time, we included documented mycobiome data into a version of the ecological Climax/Attack model that opens new lines of thoughts about the physiopathology of CF lung disease and future perspectives to improve its therapeutic management.

## Introduction

Lung infections play a critical role in cystic fibrosis (CF) pathogenesis where they can lead to significant acute decrease of lung function, known as CF pulmonary exacerbation (CFPE). Developments of next-generation sequencing (NGS) approaches allowed us to understand microbiome composition that can contribute to lung physiopathology in both healthy individuals and patients with lung disease. More recently, NGS together with advances into statistical network inference tools allowed to analyze the microbial airway communities, appreciate the inter-kingdom equilibrium of respiratory floras, and therefore develop understanding of microbial communities as a whole^1–7^.

Acute CFPEs represent major clinical events that significantly decline the lung function, contribute to disease progression and require adapted, prompt anti-infectious treatment^8–11^. Omics approaches confirmed associations between bacterial community and exacerbation^12–20^. Apart from bacteria that are well known agents causing dramatic recurrent CFPEs, respiratory viruses have been recently found to be associated with CFPE, independently of bacteria or as trigger for bacterial infection^21–23^. Fungal species such as *Candida albicans* and *Aspergillus fumigatus* have been associated with decreased lung function as well as increased frequency of exacerbations^24,25^. However, only limited number of studies evaluated mycobiome during CFPE^12,26^, and never in association with bacterial community to address inter-kingdom crosstalk.

Although much has been learned about CFPEs, our current knowledge about its pathophysiology and usefulness of diagnostic criteria remains limited. Since CFPEs are difficult to predict, new biomarkers that may derive from current NGS approaches are needed to complete existing assessments^9,13,14^. Given the prevalence of fungal colonization, the predominance of allergic bronchopulmonary aspergillosis (ABPA), the growing role of fungal infection in CF and the stabilizing function that fungi appear to play in lungs^1,27–30^, we hypothesized that fungi are involved in CF pulmonary disease evolution and in CFPEs. In the present study we considered both fungal and bacterial communities as a unique pathogenic entity, and interpreted our results according to a co-occurrence/co-exclusion pattern approach that highlights relationships between microorganisms sharing the same ecological niche, i.e. the CF respiratory tract. According to our inter-kingdom network results, we selected species belonging to *Aspergillus* and *Streptococcus* genera, and documented experimentally the predicted positive growth of *A. fumigatus* when it is co-cultured with species from *Streptococcus* mitis group. By adapting Phy-Lasso method we pointed out the fungi relevantly associated with CFPE and/or a decline in lung function. Collectively, these data provided an opportunity to reflect on a revised version of the CF Climax/Attack model (CAM) derived from ecology research field^31^. In CAM model, CF lung infection is no longer interpreted as an invasion of a single pathogen such as *Pseudomonas aeruginosa* but as dynamic changes within the whole microbial community composed of two types of microbial populations (an Attack transient but virulent population associated with CFPE and a Climax chronic population driving the long-term prognostic of CF lung disease)^4,7,13,14,31,32^. While it would be still early to draw definitive conclusions, our results pave the way for future studies on respiratory mycobiome that should help us to begin deciphering the physiopathology of CF lung disease and to improve its therapeutic management. They also suggest the complexity of all respiratory microbiome components’ connections, which might yield important insights within the CF disease.

## Results

### Patients and sample characteristics

Among the 37 patients initially screened, samples of patients 2, 7, 28 and 35 were excluded because their corresponding rarefaction curves and/or numbers of sequences were inadequate to allow biodiversity comparison. Samples from 33 patients, corresponding to 246,228 and 304,914 bacterial and fungal reads respectively, were used to evaluate microbial composition, diversity, and perform network analysis. Patients’ characteristics, spirometry data, presence of conventionally cultured pathogens in sputum, and therapeutic management at sampling time are recorded in Table 1. Seventeen patients were classified as having CFPE while 16 were not. According to body mass index (BMI), Shwachman-Kulczycki score (S-K score), and spirometry data (in particular Forced Expiratory Volume in 1 s (FEV1) expressed as percent of predicted value), patients were in a medium to advanced stage of CF disease, with several co-morbidities (exocrine pancreatic dysfunction in 90% of cases, and diabetes mellitus or impaired glucose tolerance in 33% of cases). Colonization by *Pseudomonas aeruginosa* was highly prevalent (96.7%, Table 1); all excepted one patient (a 23-year-old woman, patient 14) had positive mycological cultures. *Candida albicans*, other *Candida* sp., and *A. fumigatus* were observed in 66.6%, 18.2%, and 66.6% of patients respectively, as determined by conventional culturing. Most of the patients received rhDNAse and/or inhaled steroids (100%), low dose of azithromycin (69.7%) and antibiotics (90.1% of cases administered orally). Antifungal therapy was prescribed for one patient in agreement with ABPA diagnosis^33^. While 11 (33%) patients were treated with systemic steroids, no other systemic immunosuppressive therapy was used. No significant difference for those variables was observed between patients with and without CFPE, except for the median age, which was significantly lower in patients with CFPE.

**Table 1:** Patients’ characteristics at inclusion.

Characteristics of patients	Exacerbated N = 17	IQR^*^	Stable	IQR	p-value
**Demographic data**					
Median Age in years	23	22–30	35	29–36	<0.01
Ratio Male/Femme	1.5		1.6		NS^**^
**Clinical data**					
Median Sweat chloride test (mmol/L)	129 (n = 8)^***^	126–138	109	86–130	0.17
Median S-K Score	75 (n = 16)	53–81	73 (n = 14)	65–80	0.5
Median BMI (Kg/m^2^)	19.9 (n = 16)	18.2–19.7	19.8 (n = 15)	18.2–20.3	0.61
Median FEV1 (%)	49	41–58	55	41–60	0.72
**FEV1 percentage predicted value**					
Mild FEV1 (>70%)	9		4		NS
Moderate FEV1 (40–70%)	4		8		NS
Severe FEV1 (<40%)	4		4		NS
CTFR mutations (ΔF508 homozygous)	7 (n = 16)		2 (n = 15)		NS
**Microbiological data**					
**Positive bacterial sputum cultures for:**					
Methicillin-sensitive *Staphylococcus aureus*	4 (n = 16)		2 (n = 14)		NS
*Stenotrophomonas maltophilia*	1		1		NS
*P. aeruginosa* non-mucoid strain	7		5		NS
*P. aeuruginosa* mucoide strain	8		9		NS
**Positive mycological sputum cultures for:**					
*C. albicans*	12 (n = 16)		10 (n = 14)		NS
Non-albicans *Candida*	2		4		NS
*A. fumigatus*	12		10		NS
*Scedosporium sp*.	1		1		NS
*Penicillium sp*.	1		6		NS
**Therapeutic data**					
**Treated with:**					
Nebulized recombinant human DNase (rhDNAse)	17		16		NS
Azithromycin (sub-therapeutic dosage)	15		8		NS
Inhaled steroids (continuous)	17		16		NS
Systemic corticoids (continuous or as bolus)	8		3		NS
Intermittent antimicrobial agent exposure^****^					
**Oral antibiotics**					
0, 1 to 3 regimens	14		15		NS
>3 regimens	0		1		NS
Continuous	3		0		NS
Oral anti-fungal treatment	1		0		NS

*Values are indicated in median values (and interquartile range) for continuous data and number of patients for categorical data; ^**^NS: No significant difference; ^***^If missing values are present, the effective numbers of patients are shown in parentheses; ^****^Trimethoprim-sulfamethoxazole not included.

### FEV1 was associated with changes in alpha diversity, and CFPE with shifts in minority populations

**Figure 1:**
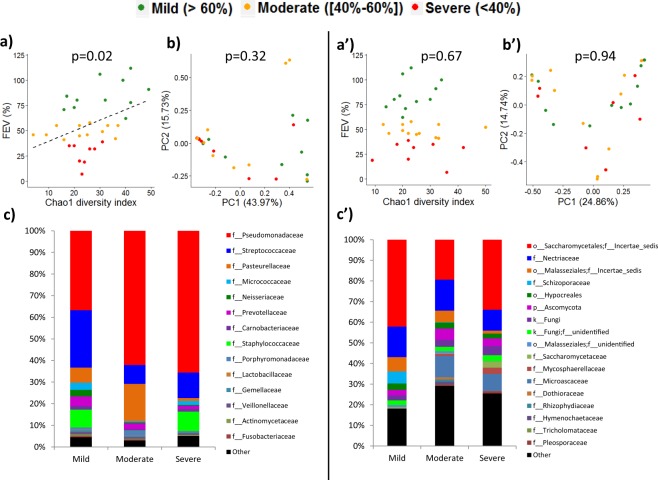
Diversity and taxonomy summaries of sputa collected from CF patients with a mild (), moderate () and severe () alteration of the lung function measured by FEV1. Dataset from each sputum sample contained an average of 7,909 bacterial reads (ranging from 5,104 to 14,749), and an average of 9,811 fungal reads (ranging from 2,933 to 15,599). Alpha diversity indexes of the bacterial microbiome (**a**) but not of mycobiome (**a′**) were positively correlated with FEV1 values. When patients were divided into 3 groups according to FEV1 values^59,71^, bi-dimensional PCoA representations (**b**,**b′**) based on Bray-Curtis similarity matrix did not show clustering between groups. However, we observed a high proportion (62% and 66%) of Pseudomonadaceae (mainly composed of *Pseudomonas* species) among patients exhibiting a moderate and severe disease, while it represents only 32% of the bacterial composition among patients with a mild ventilatory deficit (**c**). The fungal composition exhibited also some shifts, especially a decrease in OTUs belonging to Malasseziales in samples from patients with severe lung decline (**c′**). Taxonomy composition were represented at family level of bacterial (**c**) and fungal (**c′**) microbiotas; fungal reads that were not identified as family levels are grouped at order or phylum levels.

Rarefied data was used to summarize microbial composition and its changes among groups (Fig. 1). These microbial communities inferred from NGS were consistent with results obtained by culturing (Table 1); however greater diversity was evident via NGS approach. We observed a significant positive linear relationship between FEV1 values and Chao1 indexes of bacterial communities (Fig. 1a). This linear relationship was also observed when using Shannon diversity indexes (p = 0.04), but not using Simpson diversity indexes (p = 0.10). Different diversity metrics capture, indeed, different information. Microbial populations were divided into majority and minority ones^12,34^. Microbiota and mycobiota of patients with CFPE exhibited a significant decrease in *Haemophilus*, *Neisseria*, and *Cladosporium* genera among majority populations, as well as changes in relative abundances of bacteria significantly associated with a loss in alpha diversity of fungal and bacterial minority populations (Table 2). To evaluate better inter-kingdom equilibrium between the microbial communities, we applied a ratio of fungal to bacterial diversity previously reported in IBD^35^. Fungi-to-bacteria diversity ratios showed a dominance of bacteria on fungus with a median at 0.64 (IQR = [0.43; 4.25]), without any significant disequilibrium between bacterial and fungal loads among patients with and without CFPE. Median of the Basidiomycota/Ascomycota relative abundance ratio was 0.03 (IQR = [0.01; 0.19]).

**Table 2:** Alpha diversity and abundance differences of total, minority and majority microbial populations among patients without and with CFPE.

	Total Population			Minority Population			Majority Population		
Patients	Without CFPE	With CFPE	p-value	Without CFPE	With CFPE	p-value	Without CFPE	With CFPE	p-value
**Alpha diversity (score)**									
Bacterial composition:									
Shannon index	0.7 [0.2;1.4]	0.4 [0.3;1.5]	NS	2.2 [2.1;2.4]	2.2 [1.8;2.4]	NS	0.6 [0.2;0.8]	0.4 [0.2;1.0]	NS
Simpson index	0.3 [0.1; 0.5]	0.2 [0.1;0.6]	NS	0.8 [0.8;0.9]	0.8 [0.8;0.9]	NS	0.3 [0.1;0.4]	0.1 [0.1;0.5]	NS
Chao1 index	27.0 [21.0;36.3]	23.0 [20.0;30.5]	NS	19.5 [13.0;28.5]	16 [14.5;23.0]	NS	7.5 [7.0;8.0]	6.0 [6.0;7.5]	NS
Fungal composition									
Shannon index	1.5 [1.1;1.9]	1.4 [1.2;1.7]	NS	1.5 [1.4;1.7]	1.0 [0.8;1.5]	*	1.2 [1.1 ;1.5]	1.1 [0.9 ;1.4]	NS
Simpson index	0.7 [0.5;0.8]	0.6 [0.5;0.8]	NS	0.7 [0.6;0.8]	0.5 [0.4 ;0.7]	*	0.6 [0.5 ;0.7]	0.5 [0.5 ;0.7]	NS
Chao1 index	23.5 [20.0;28;3]	20.0 [17.5;24.0]	NS	12.0 [8.8;16.5]	10.0 [9.0 ;14.0]	NS	11.0 [10.0 ;12.0]	9.0 [7.0 ;11.0]	**
**Relative abundance (%)**									
Bacteria									
*Haemophilus*	0.8 [0.3;7.2]	0.0 [0.0;0.5]	**	—	—	—	0.0 [0.3;7.4]	0.0 [0.0;0.6]	**
*Neisseria*	0.1[0.0;0.8]	0.0 [0.0;0.0]	*	—	—	—	0.1 [0.0;0.8]	0.0 [0.0;0.0]	*
*Scardovia*	0.0 [0.0;0.0]	0.0 [0.0;0.1]	*	0.0 [0.0;0.0]	0.1 [0.0;3.5]	*	—	—	—
*Megasphera*	0.0 [0.0;0.1]	0.0 [0.0;0.0]	**	0.0 [0.0;0.5]	0.0 [0.0;0.2]	*	—	—	—
*Kingella*	0.0 [0.0;0.0]	0.0 [0.0;0.0]	**	0.0 [0.0;0.2]	0.0 [0.0;0.0]	**	—	—	—
*Leptotrictia*	0.1 [0.0;0.2]	0.0 [0.0;0.2]	NS	3.2 [1.7;5.9]	0.8 [0.0;2.5]	*	—	—	—
*Clostridia*	0.1 [0.0;0.1]	0.0 [0.0;0.0]	**	0.0 [0.0;0.6]	0.0 [0.0;0.0]	*	—	—	—
*TM7*	0.1 [0.0;0.1]	0.0 [0.0;0.1]	**	1.2 [0.1;3.0]	0.0 [0.0;0.8]	*	—	—	—
Fungus									
*Cladosporium*	0.0 [0.0;0.7]	0.0 [0.0;0.0]	**	—	—	—	0.0 [0.0;0.8]	0.0 [0.0;0.0]	**

(NS p ≥ 0.10, *p < 0.0.05, **p < 0.0.01).

### Inter-kingdom correlation and network analysis

**Figure 2:**
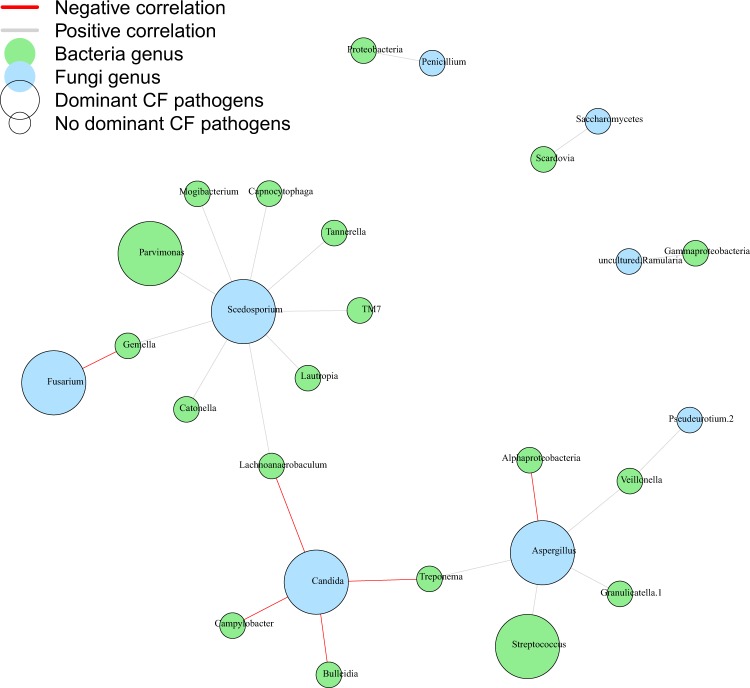
Co-occurrence network between bacteria and fungi from the ReBoot method. Bacteria and fungi are represented by blue and green circles, respectively. The large circles refer to recognized dominant CF pathogens. Gray lines connecting circles represent strong positive correlation, while red lines represent strong negatively correlation. A negative correlation between a bacteria genus and a fungus genus refers to an increase of bacteria genus abundance and a decrease of fungus genus abundance; a positive correlation to an increase of bacteria genus abundance and an increase of fungus genus abundance.

**Figure 3:**
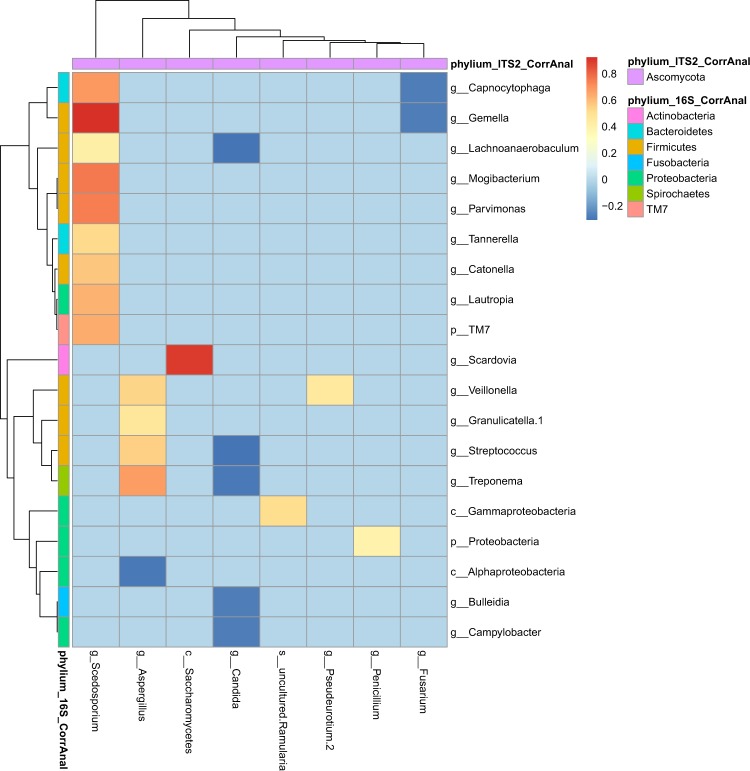
Inter-kingdom correlation from the ReBoot method. Statistical significance was determined for all pairwise comparisons; only significant correlations (p < 0.05) are displayed. Warm colors (red to yellow squares) indicate positive correlations, and cool colors (dark blue to light blue squares) indicate negative correlations.

We analyzed co-presence or absence patterns between bacteria and fungi, by building a correlation matrix at genus levels of the whole set of NGS data and representing it as a graphical network (Figs. 2 and 3). We inferred an inter-kingdom network by plotting bacterial genera significantly correlated with at least one fungal genus and vice versa. The network was composed of 26 nodes and 23 edges and it revealed three main clusters organized around *Aspergillus*, *Candida* and *Scedosporium* genera plus three disconnected pairs of microorganisms (Fig. 2). Among these pairs, two contained environmental fungi: *Ramularia* (a phytopathogen) and *Penicillium* connected with the class of Gammaproteobacteria and the phylum of Proteobacteria, respectively. These fungi likely represent environmental transitient fungal elements, as previously proposed^12,36,37^. Among the six negatively correlated OTUs identified in our model, *Fusarium* sp. appeared to display a negative correlation with *Gemella* sp., the latter identified as keystone genus predicting CFPE^4,13^. The other positively correlated OTUs predict interactions between *Aspergillus*, *Candida*, or *Scedosporium* and bacteria belonging to *Capnocytophaga*, *Parvimonas*, *Streptococcus*, or *Veillonella*. Apart from *Scedosporium* species that were described as belonging to Climax population^31^, our network results preferentially associate fungi to the bacterial population identified during CFPE and referred as Attack population in the ecological Climax/Attack Model (CAM) recently proposed^4,13,31^.

### Assessment of network interactions using *in vitro* co-cultures

**Figure 4:**
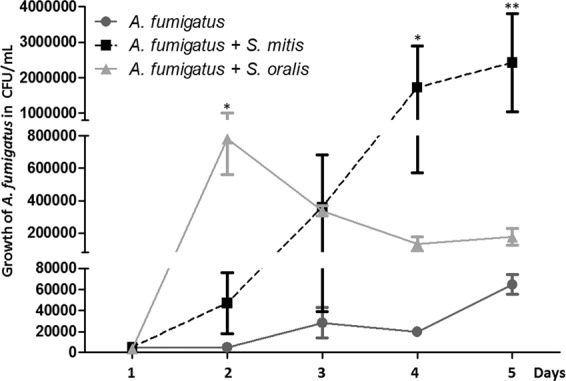
Growth curves for *in vitro* co-cultures of *A. fumigatus* with *S. mitis*, or *S. oralis* compared to culture of *A. fumigatus* alone. *A. fumigatus* growth () expressed in CFU/mL is significantly enhanced by *S. mitis* at day 4 (p < 0.05) and day 5 (p < 0.01). Growth of *A. fumigatus* plus *S. mitis* () is significantly higher than growth of *A. fumigatus* plus *S. oralis* () at day 5 (p < 0.05). Growth of *A. fumigatus* was not significantly enhanced by *S. oralis* excepted at day 2 (p < 0.05).

To document inter-kingdom interactions that we identified in correlation matrix analysis, we performed co-culture experiments between *A. fumigatus* and *Streptococcus mitis* or *Streptococcus oralis*. We selected these microorganisms because of their well-established medical importance in CF (at the species level), high quantity in our cohort associated with a robust positive co-variation (at the genus level), and known conditions for *in vitro* cultures. As predicted, *A. fumigatus* growth appeared to be enhanced by both *S. mitis* and *S. oralis* (Fig. 4). As our experimental method was focused on *A. fumigatus* growth that has a duplication time clearly different from *Streptococcus* sp. (24–48 h for *Aspergillus* versus about 1 h for *Streptococcus*), it could not inform on bacterial growth.

### Microbiome and mycobiome markers of CF lung dysfunction

**Figure 5:**
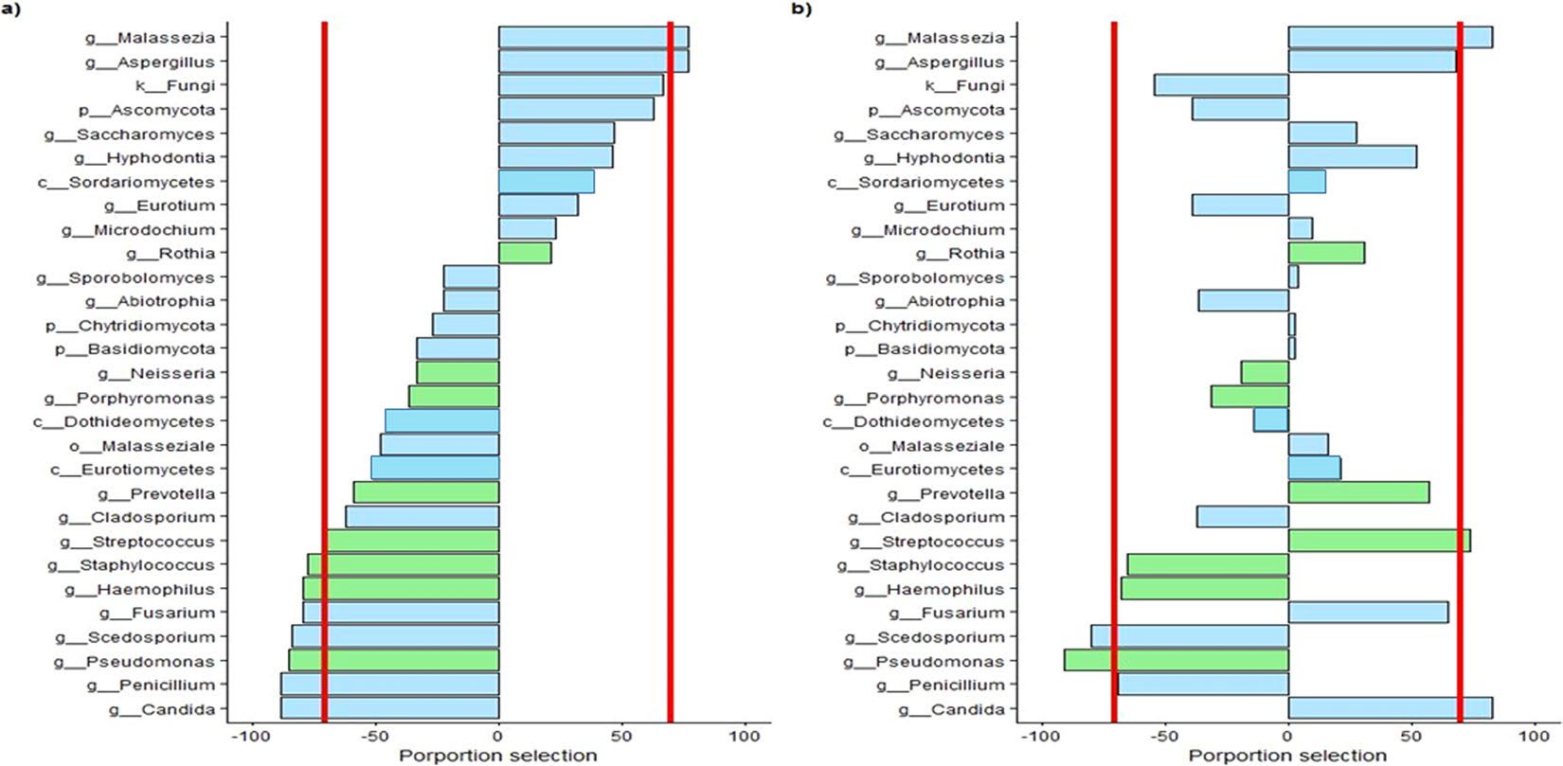
OTUs relevantly associated with CFPE (**a**) and FEV1 (**b**) by bootstrap-enhanced Phy-Lasso. Only genera of bacteria (in green) and of fungi (in blue) selected with a frequency ≥15% are represented. Bars are represented at left when the genus was negatively associated, and at right when the genus was positively associated with CFPE or FEV1. We considered OTU consistently associated to clinical features when its selection frequency was greater than 70% (red lines).

**Figure 6:**
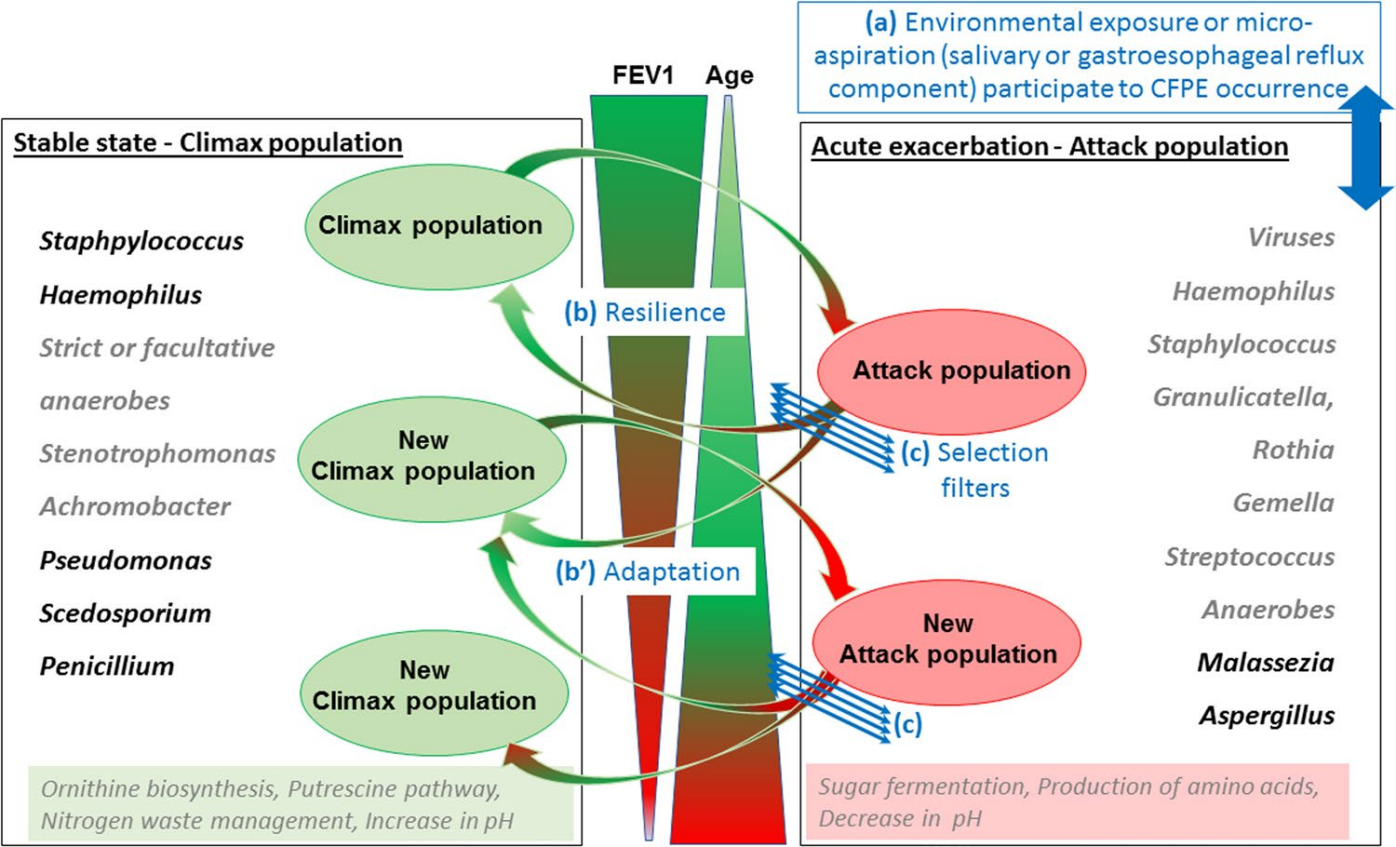
Adaptation of Climax/Attack model (CAM) in CF as inferred from previous studies and our study^4,7,13,14,31,32^. According to CAM, two different microbial populations are evolved dynamically in CF lungs, both being potentially composed of anaerobes^4,7,13,14,31,32^. Microbiome changes acquired through environmental exposure or salivary aspiration (**a**) seem to participate to CFPE occurrence leading to an Attack population. The microbial community will return to its original state (Resilience in (**b**)) or move to a new community considered as stable but composed of a new Climax population with a different microbial community (Adaptation in (**b**)), according to perturbation forces of the Attack population and its ability to pass through selection filters (**c**). Selection filters (**c**) refer to layers that influence evolution of the microbial structure: changes in nutrient, sources-oxygen pressure, pH, level of microorganism growth and virulence, host immune and inflammatory response, antimicrobial treatment pressure. They participate in selecting the population the most adapted to the new airway remodeling, in a circular relationship. According to a microbial community partitioned by carbon source, the Climax population uses amino acids and produces ammonia, while Attack population uses sugar fermentation and produces acid. In this context, *Malassezia* yeasts which use lipids as carbon sources are unable to ferment sugar, and may have some advantages (cross-feeding) to share ecological niche with anaerobes and *Streptococcus*. *Streptococcus* are responsible for sugar fermentation producing amino acid. By the same time, fermentation is decreasing pH that is favorable for expansion of anaerobes which contribute to produce fermentative compounds. Furthermore, *Malassezia* are able to develop at acid pH. On the other side, *Scedosporium* are associated with decline of FEV1, which fit well with a role into an advanced Climax population in agreement with their ability to use a wider range of nutritive substrates, to synthesize specialized metabolites including ammonia fermentation, and their high resistance to antifungal drugs. Genera in black refer to our bootstrap-enhanced Phy-Lasso analysis, and genera in grey to previous CAM studies.

To fully predict the bacterial and fungal communities associated with altered lung function, we applied a feature selection method (bootstrap-enhanced Phy-Lasso) to identify genera relevantly associated with CFPE (Fig. 5a) and/or FEV1 decline (Fig. 5b). In patients’ microbiomes *Haemophilus*, *Pseudomonas*, *Staphylococcus*, *Streptococcus*, *Candida*, F*usarium*, *Penicillium*, and *Scedosporium* were selected 150 times out of 200 bootstrap replicates and were negatively associated with the presence of CFPE. *Malassezia* and *Aspergillus* were positively associated with CFPE (Fig. 5a). *Pseudomonas*, *Penicillium*, and *Scedosporium* were negatively associated with FEV1, an increase in their relative abundance being associated with a decrease in FEV1 measure (Fig. 5b). Altogether, these results highlighted the complexity of the microbial community interacting within the CF respiratory tract, and suggested the suitability of developing ecological models such as CAM. Collectively, our findings (inter-kingdom network analysis, *in vitro* co-culture results, and feature selection based on bootstrap-enhanced Phy-Lasso) pave the way for deciphering the role of fungi in CF lung disease at the ecological level by proposing a new version of the recently-described CAM model^31^ (Fig. 6).

## Discussion

CFPEs represent key intermittent events in CF disease that are poorly defined but clearly associated with the decline in lung function and accelerated disease progression. They require specifically adapted anti-microbial treatments^8–11^ but those treatments fail to recover lung function of 25% of patients^38^. Therefore omics approaches represent promising tools to identify and characterize relevant biomarkers able to predict CFPE and to improve therapeutic monitoring^13^. Considering that CF respiratory tract is polymicrobial, i.e. composed of bacteria, viruses and fungi, and since all these microorganisms are able to induce CFPEs, the large data set generated by omics methods should take into account all resident lung microorganisms. Here, we performed a combined analysis of CF airway microbiome and mycobiome, from the microbial diversity to the inter-kingdom network investigation in relation with patient clinical status.

We reported a decrease in alpha diversity of bacteria associated with FEV1 decline and, in agreement with published data^12,14–17,19,20,26,36,37,39–44^. We identified *Pseudomonas*, *Streptococcus*, *Haemophilus*, *Candida*, *Fusarium*, *Penicillium*, and *Scedosporium* as dominant genera (Fig. 1) and numerous shifts in both fungal and bacterial minority populations during CFPE (Table 2). Similarly, Cuthbertson *et al*.^42^ evidenced a greater turnover rate within the rare species compared to the microbial core. Moreover, the simultaneous analysis of mycobiota and microbiota allowed us to appreciate the inter-kingdom interactions. The observed ratios of fungus-to-bacteria diversity were 10 times higher compared to the published digestive ratios^35^, supporting the notion that lung mycobiome is derived from inhaled spores and transient species^12,36,37,45^. Our estimation of the Basidiomycota/Ascomycota relative abundance ratios was lower than digestive ones^35^ revealing the excess of Ascomycota, the phylum to which fungal pathogens belong. Together, these results suggest a colonization of airways during CF following an exposure to fungal spores.

Our study design has some notable limitations: *(i)* First, even though the clinical features of our CF population (Table 1) are congruent with published data^21,26,27,37,39,44^, the limited cohort we analyzed retrospectively was based on samples stored at −20 °C, which did not allowed us to realize RNA extraction and meta-transcriptomic analysis. *(ii)* Secondly, we used sputum samples, which could be contaminated with a buccal flora. However, this sample type is easy to assess, not invasive compared to bronchial alveolar lavage and sufficiently robust in predicting microbiome as previously demonstrated^7,39,46^. *(iii)* The small sample size (as for the majority of studies about the association between respiratory microbiota or mycobiota and CF^12,26,44,47^) decreased the statistical power to recognize small differences if present. *(iv)* Finally, the complex structure and the high-dimensional data generated by NGS analysis required specific statistical methods and computational tools that have to be carefully evaluated. We acknowledge that although correlation does not indicate causation; it can provide a reasonable starting point to assess inter-kingdom network, especially when supported by clinical knowledge about pathogenic species (Figs. 2 and 5) and experimental *in-vitro* corroboration (Fig. 4). For the first time, the mycobiome aspect of the CAM model recently adapted from the ecology field^4,31^ is conjectured from CF fungal data (Fig. 6).

Niche-microbe interaction models have been developed to better interpret the dynamics, composition and role of lung microbial communities^4,7,31,48–50^. These models were focused on bacterial communities and suggested that relative abundances of bacteria were not driven by a neutral model of dispersion in CF^50^. In CF respiratory tract, microbiome assembly is resulting from dynamic selective pressures that are changing with each stage of lung disease. In different layers characterized by local nutrient availability, pH, oxygen pressure, host immune response and antibiotic treatment, a selection of microbiome members occurred and consequently a shift in microbial composition. So far, only CAM model has attempted to formally describe the lung mycobiome^31^.

Even if the role of anaerobes is still matter of debate in terms of community composition, metabolome, and resistome^51,52^, two types of bacterial populations have been highlighted in CAM model. These two populations have distinct metabolic potential defined by carbon source: an Attack transient but virulent population associated with CFPE and a Climax chronic population driving the long-term prognostic^4,7,13,14,32^. While anaerobes have been first affiliated to Climax population^31^, the Attack community appeared to be composed of anaerobes (*Prevotella*, *Parvimonas*, *Veillonella*, *Porphyromonas*, and *Fusobacterium*), facultative anaerobes (*Streptococcus*, *Granulicatella*, and *Rothia*), and the obligatory fermentative (*Gemella*) that have been associated with fermentation pathway producing organics acids and decrease in pH^4,7,13,14,32^. The Climax community is composed of *Pseudomonas*, *Staphylococcus*, *Stenotrophomonas*, and *Achromobacter* that were strongly associated with ornithine biosynthesis, transport of putrescine, and that were producing ammonia and an increase in pH^4,13^. Taking into account this model^4,7,13,14,32^ and our results, data allowed us to make small steps toward achieving a revised version of CAM model in the CF context (Fig. 6), in which the mycobiome role is completed as follow.

The decline in lung function in CF is driven by clinical changes especially by CFPEs, which require active treatment to recover from CFPE and go back to a steady state that may include chronic colonization^4,19,20^. The triggering elements of state shifts are not well known, but it has been proposed that microbiome changes acquired through environmental exposure or aspiration participate to CFPE occurrence leading to the Attack population ((a) in Fig. 6)^31,53^. Unexpectedly, both *Staphylococcus* and *Pseudomonas* appeared to be negatively correlated with CFPE (Fig. 5). This result may be explained by a CF population composed of adult patients with a rather mild to moderate lung diseases than a severe one (Table 1). Besides being involved into CFPE, a recent interest research has emerged in determining whether *Staphylococcus aureus* can have both damaging and protecting effects for the CF host according to the differences phases in the evolution of the CF airways infection^54^. Our results (Fig. 5b) agree with earlier studies in which colonization with *P. aeruginosa* and methicillin-resistant *S. aureus* was strongly associated with a loss of lung function (a loss of FEV1) in CF^55^ while microbiota communities appeared to not differ based on pulmonary exacerbation status of CF patients^39,56^. After CFPE occurred, the perturbed microbial ecosystem will return to its original stable state (Resilience (b) in Fig. 6) or if the perturbation is not controlled, will evolve to a new ecosystem considered as stable but composed of a novel Climax population that differs from the previous community (Adaptation (b’) in Fig. 6). According to the CFPE severity, different filters (changes in nutrient sources, level of microorganism growth and virulence, strong host innate immune and inflammatory response, antimicrobial treatment pressure (c) in Fig. 6) will select this new population well adapted to the airway remodeling, in a circular relationship. As microbial community composition and disease progression proceed hand-in-hand, assembly of CF microbiome and mycobiome will result from dynamic events, especially from competitive exclusion or co-occurrence events observed through OTU network and related functional analysis^4,13^. In agreement with the bacterial community partitioned by carbon source^4^, our results suggest fungal species preferentially as Attack members (Fig. 5), which is consistent with clinical and mycological data^28–30^. Among them, *Aspergillus* and *Malassezia* were significantly associated with CFPE (Figs. 5a and 6). This result is in agreement with the ability of *Aspergillus* to be responsible for ABPA and related exacerbations in CF^28,29^. It goes in the same direction of previous results demonstrating an increased risk of CFPE requiring hospitalization when patients are colonized with *A. fumigatus*^25^. As *Aspergillus* was previously assigned to Climax population^31^, our findings have to be confirmed in a larger CF population. *Malassezia* are lipophilic yeasts, and require specific culture conditions that are not routinely used when a pulmonary exacerbation is monitored. Therefore, *Malassezia* have not been associated with CFPE yet. However, they have been identified as biomarker of chronic inflammatory diseases such as asthma^57^, or inflammatory bowel disease^35^. It may reflects a clear advantage (cross - feeding) that these yeasts get in sharing ecological niche with anaerobes and *Streptococcus*, since they produce multiple secreted lipases that may help in recycling lipids produced by fermenting bacteria^13,58^. In addition, *Malassezia* -which are part of the normal skin flora- are able to develop at acid pH (skin surface pH is below 5).

On the other side, *Scedosporium* were significantly associated with a decline in FEV1 (Fig. 5b), a result congruent with their ability to induce chronic detrimental colonization of CF lung during Climax phases^12,14,15,17,19,20,42,59–63^. As *Pseudomonas* do, *Scedosporium* is able to produce secondary metabolites with antimicrobial activity^64^. It secretes a polyketide boydone A with an anti-staphylococcus activity against methicillin-resistant isolates, which fits well with the course of microbial airway colonization clinically observed in CF. Moreover, the recent whole genome sequencing of *Scedosporium* species (https://genome.jgi.doe.gov/programs/fungi/index.jsf) revealed a number of genes encoding putative ankyrin motif-containing proteins, methyltransferases and oxidoreductases larger than the one of *A. fumigatus* genome^65^. Whether this larger magnitude of activities allows the mold to use a wider range of nutritive substrates or to be able to synthesize specialized metabolites including ammonia fermentation remains to be elucidated^64–66^, but it may participate in its successful establishment within the respiratory tract of CF patients. Over time, the interchange of these states will lead to the emergence of new Climax communities that are more and more adapted to the airway remodeling, leading to a less diverse community, composed of microorganisms highly resistant to antibiotic or antifungal agents such as *Pseudomonas* or *Scedosporium*.

In summary, microbial interactions are clearly complex in natural communities such as lungs, due to the impact of a multitude of microbes (bacteria, fungi, but also phages and viruses) able to interact^67,68^ and to host immune response that appears dual^68,69^. We show that NGS approach combined with inference network and ecological model analysis are useful in helping to decipher physiopathology of CF lung disease, and can be considered as a promising tool for improving our therapeutic protocols. As we clearly improved our knowledge on sequenced genomes of the most frequent molds in CF (https://genome.jgi.doe.gov/programs/fungi/1000fungalgenomes.jsf), such inter-kingdom analysis should be easily reproduced in a larger cohort, completed to include all significant members of the CF lung microbial community, and extended to analyze the meta-transcriptome and metabolome, in order to confirm or to correct the CAM model efficiency and to define accurate biomarkers of CFPEs. Additional *in vitro* (cell cultures) and/or *in vivo* (animal) models are required to support our future findings and to fully understand the complex microbial interactions that drive lung microbial community evolution in CF.

## Materials and Methods

### Patients and sputum samples

Among CF patients enrolled into a 3-year multicenter national prospective study (PHRC number 06/1902, acronym: MucoFong), 37 sputa consecutively collected at hospital centers of Lille (n = 19), Dunkerque (n = 3), and Grenoble (n = 15) were sequenced using NGS. They were alternatively selected from the first 64 sample collection^21^ according to CFPE presence or absence, if spirometry, therapeutic, radiological and biological data were collected, and if residual sputum sample was sufficient to perform DNA extraction. The clinical status (pulmonary exacerbation or stable period) of each patient was defined by the physician as follow. Recent changes in clinical parameters and/or modifications of the pulmonary function provide criteria of exacerbation according to European Respiratory Society statement^10^.

Sputa were collected and analyzed according to a standardized protocol previously described^21,70^. DNA extraction was performed as previously described^21^.

### Ethics statement

MucoFong study was approved by the institutional ethics committees of Lille Hospital (Reference Number: CPP-06/84), and a written informed consent was provided by all participants. All methods were performed in accordance with the relevant guidelines.

### Targeted metagenomic library preparation, sequencing and taxonomic assignation

V3-V4 and ITS2 regions of bacterial and fungal rDNA were amplified and sequenced as previously described^37,44^. All reactions were prepared in a sterile PCR hood, and negative control reactions were performed and examined by electrophoresis on 1.5% agarose gel. We obtained 429,565 and 443,270 pyrosequences with prokaryotic and eukaryotic loci, respectively. Sequencing data processing was performed using the Metabiote© pipeline where potential chimeras, read with poor quality (≤20 quality scores), short read (≤150 nucleotides for 16S rRNA gene, ≤100 nucleotides for ITS2 rRNA gene), homopolymers, singletons, and doubletons were removed before the creation of Operational Taxonomic Unit (OTU). OTUs were created applying complete-linkage clustering with 97% similarity criteria (UClust v1.2.22q); OTUs’ annotation was performed using Ribosomal Database Project classifier against Greengenes (v13.8 containing 203,452 16 S rRNA sequences) and UNITE (v12.11 containing 97,868 ITS1-ITS2 sequences) databases (Supplementary Table 1). Rarefaction curves were calculated to determine whether deep sequencing was sufficient to accurately characterize the bacterial and fungal community diversity, as previously described^44^.

### Microbial community composition analysis

Bacterial and fungal data were analyzed at phylum to species levels. Clinical status was assessed using CFPE (patient acutely exacerbated or stable), S-K score, BMI, and the respiratory capacity measured by FEV1 value. The later was also expressed as categorical by using cut-off values: “mild” (FEV1 > 70%), “moderate” (FEV1 between 40–70%) and”severe” ventilatory deficit (FEV1 < 40%)^59,71^. Microbial composition was analyzed according to clinical status measured as both continuous and categorical variables.

Alpha diversity was measured though Simpson, Shannon, and Chao1 indexes. Richness and evenness differences between clinical status groups (S-K score, BMI, and FEV1) were tested using Wilcoxon signed-rank test. Since these alpha diversity indexes do not reflect accurately community composition (i.e. two communities could have the same diversity index but different community structures), principal coordinate analysis (PCoA) of beta diversity analysis was performed using Bray-Curtis distance. Structural differences between groups were tested using analysis of dissimilarities (ANOSIM) test. Bacterial and fungal community diversities were analyzed through the vegan and fossil R packages (v 3.4.3). Their structures were analyzed using QIIME (v 1.9.1) software and vegan package. To assess inter-kingdom equilibrium between the microbial communities, we computed a fungus-to-bacteria diversity ratio and a Basidiomycota-to-Ascomycota relative abundance ratio^35^ that were compared between patient groups using Wilcoxon signed-rank test. Relative abundances of OTUs were compared between groups as follow: for each feature (be OTU, genus, etc.) present in at least 10% of the patients, we first tested the distributional adequacy of the Poisson, the negative binomial and the Gaussian to the normalized non-zero data. Secondly, we considered the model presenting the best fitted distribution and we tested the differences between groups. When none of the three distributions fitted, Wilcoxon signed-rank test was used. Goodness-of-fit tests for discrete distributions were implemented through the vcd package (v 1.4.4). The Shapiro-Wilk test was used to test for normality as well as graphical check. Bioconductor packages DESeq. 2 (v 1.22.1) and edgeR (v 3.16.1) implemented differential analysis for the model with discrete distributions, while metagenomeSeq package (v 1.18.0) implemented differential analysis for the zero inflated Gaussian model^72^. Finally, each sample was divided into majority and minority populations (abundant population referring to an OTU relative abundance greater than or equal to 1%, and minority population as having a relative abundance of less than 1%), since their contribution may differ^12,34^. According to Carmody *et al*.^15^, we defined a dominant OTU as the most abundant OTU having a relative abundance higher or equal than twice the relative abundance of the second most abundant OTU for each sputum sample. We compared both abundant and minority populations as well as dominant OTUs between clinical status groups.

### Inter-kingdom network analysis

To assess the relationships among microorganisms and to determine their underlying physio-pathological senses, joint analysis of fungal and bacterial relative abundance data was performed by estimating their correlation matrix.

According to Gevers *et al*.^73^, to test the correlation while accounting for the compositional nature of data (i.e. proportions that sum to 1), the permutation-renormalization bootstrap method (ReBoot) was used. We built both the null distribution of correlations from renormalized permuted data which represents the correlation structure arising purely from the compositionality, and the distribution of correlations from bootstrap sampled data referring to the confidence interval of the observed correlation. The compositional null distribution and the bootstrap distribution were compared by Z-test with the variance pooled from both distributions. The ccrepe Bioconductor package (v 1.10.0) implemented this ReBoot method, and was used here; it also allows applying network analysis up from 20 samples. The significance of association between features was based on the Spearman correlation as similarity measure (at the 0.05 level). We generated 1,000 bootstrap replicates.

### *In vitro* co-cultures

*A. fumigatus* strain CBS144.89, and two clinical strains of *S. oralis* and *S. mitis* isolated from two CF patients and kindly provided by Pr. Lehours (Bacteriology Department, Bordeaux hospital) were used for *in vitro* experiments. These two Streptococcus isolates were selected according to their prevalence in CF upper airways and their role in CF inflammation and disease^74–77^. Co-cultures (10 million bacterial cells plus 1 million *Aspergillus* conidia) were conducted in brain-heart infusion (BHI) medium, with moderate shaking, at 37 °C, under aerobic conditions as recently described^1^. Growth of *A. fumigatus* alone or in co-culture with *S. oralis* or *S. mitis* was measured every 24  hours for five consecutive days. At each time point, a diluted aliquot of microbial BHI-mixture was plated on chocolate agar; fungal colony forming units (CFUs) were counted after a 24-h incubation period. The experiment was done in triplicate.

### Identification of taxonomic traits independently associated with the clinical outcomes

Among a high number of OTUs (greater than the number of patient that we assimilated to a high-dimensional dataset), we determined which ones were significantly associated with clinical status by applying Least Absolute Shrinkage and Selection Operator (Lasso) regression^78^, after adjusting for the contribution of the other OTUs. Lasso linear or logistic regression models were used when the clinical status was measured, respectively, as a continuous (by FEV1) or binary (by CFPE) variable. In Lasso regression, a penalty on absolute coefficient size is added to the usual loss functions used for regression problems (mean squared error in the linear model and negative logistic log likelihood in the logistic model). In one hand, the introduction of a penalty often improves the prediction accuracy due to the bias-variance trade-off. On the other hand, if the amount of penalty is sufficiently large, some coefficients are shrunk to exact zero, thus estimation and OTU selection are simultaneously achieved. The Lasso technique has been adapted to hierarchical data. Rush *et al*.^3^ recently proposed a Phy-Lasso method that incorporates the phylogenetic tree structure characteristic of microbiome OTUs. This Phy-Lasso method applied a hierarchical model for each taxonomic level; the corresponding code being available from the author^79^. We added Phy-Lasso linear regression to the toolbox, and estimated the optimal amount of penalty from the data as follow. Because of the small sample size of the present study, we used leave-one-out cross-validation (LOO-CV) in which the model is fitted for n-1 patients and the predicted clinical status for the left-out patient is compared with his/her actual clinical status. This procedure is repeated n times, and the average agreement of the predicted and observed clinical status computed. Among values varying between high and low amounts of penalties, the good amount of penalty is chosen such that the predicted error is minimized. In linear regression (such as FEV1), we considered the predicted quadratic error. In logistic regression (such as CFPE), we considered the predicted classification error determined from a cutoff probability of 0.5. While the Lasso has excellent properties in dimensional reduction and estimation, it over-selects OTUs to reduce the prediction errors when a cross-validation method is used. To address this point, we applied the bootstrap-enhanced Lasso (Bolasso, based on intersecting bootstrapped Lasso estimations^80^), given its appealing asymptotic consistency properties and its simple implementation. In Bolasso, only OTUs frequently chosen by Phy-Lasso over bootstrap samples are selected, which improve results stability. We generated 200 bootstrap replicates. The frequency thresh-old of Bolasso was specified to be conservative (70%), to not miss any OTU associated with clinical status. PhyLasso associated function as well as all the codes used in this study are available at: https://github.com/psBiostat/MucoFong-study.git, and summarized in the Supplementary Table 2.

### Revision of the climax-attack model (CAM) previously proposed in CF

Based on our results of inter-kingdom network analysis, *in vitro* co-cultures and feature selection based on bootstrap-enhanced Phy-Lasso, we proposed a CAM version enriched with the present mycobiome data (Fig. 6). In this new CAM version, we confirmed affiliations of bacteria previously proposed and documented^4,7,13,14,31,32^. Regarding mycobiome, we confirmed the previous *Scedosporium* genus assignation to Climax population, as it was negatively associated with both CFPE and elevated value of FEV1 (see feature selection in Fig. 5). Based on feature selection (Fig. 5), we proposed the same affiliation for *Penicillium*.

As *Malassezia* and *Aspergillus* are associated with CFPE (Fig. 5a), we proposed to affiliate these genera to the Attack population, which fit well with the positive network interaction (Fig. 2), and the co-cultures results between *Aspergillus* and *Streptococcus* (Fig. 4).

## Supplementary information

Supplementary Table 2.

Supplementary Table 1.

## References

[1] Tipton L (2018). Fungi stabilize connectivity in the lung and skin microbial ecosystems. Microbiome.

[2] O’Brien, S. & Fothergill, J. L. The role of multispecies social interactions in shaping Pseudomonas aeruginosa pathogenicity in the cystic fibrosis lung. *FEMS Microbiol. Lett*. **364** (2017). 10.1093/femsle/fnx128 PMC5812498 28859314

[3] Rush, S. T., Lee, C. H., Mio, W. & Kim, P. T. The Phylogenetic LASSO and the Microbiome. *ArXiv160708877 Q-Bio Stat* (2016).

[4] Quinn RA (2016). Ecological networking of cystic fibrosis lung infections. NPJ Biofilms Microbiomes.

[5] Kurtz ZD (2015). Sparse and compositionally robust inference of microbial ecological networks. Plos Comput. Biol..

[6] Berry D, Widder S (2014). Deciphering microbial interactions and detecting keystone species with co-occurrence networks. Front. Microbiol..

[7] Whiteson KL (2014). The upper respiratory tract as a microbial source for pulmonary infections in cystic fibrosis. Parallels from island biogeography. Am. J. Respir. Crit. Care Med..

[8] Bhatt JM (2013). Treatment of pulmonary exacerbations in cystic fibrosis. Eur. Respir. Rev. Off. J. Eur. Respir. Soc..

[9] Stenbit AE, Flume PA (2011). Pulmonary exacerbations in cystic fibrosis. Curr. Opin. Pulm. Med..

[10] Bilton D (2011). Pulmonary exacerbation: towards a definition for use in clinical trials. Report from the EuroCareCF Working Group on outcome parameters in clinical trials. J. Cyst. Fibros. Off. J. Eur. Cyst. Fibros. Soc..

[11] Goss CH, Burns JL (2007). Exacerbations in cystic fibrosis. 1: Epidemiology and pathogenesis. Thorax.

[12] Nguyen LDN (2016). Effects of Propidium Monoazide (PMA) Treatment on Mycobiome and Bacteriome Analysis of Cystic Fibrosis Airways during Exacerbation. Plos One.

[13] Quinn RA (2016). Metabolomics of pulmonary exacerbations reveals the personalized nature of cystic fibrosis disease. PeerJ.

[14] Quinn RA (2015). A Winogradsky-based culture system shows an association between microbial fermentation and cystic fibrosis exacerbation. ISME J..

[15] Carmody LA (2013). Changes in cystic fibrosis airway microbiota at pulmonary exacerbation. Ann. Am. Thorac. Soc..

[16] Tunney MM (2013). Lung microbiota and bacterial abundance in patients with bronchiectasis when clinically stable and during exacerbation. Am. J. Respir. Crit. Care Med..

[17] Zemanick ET (2013). Inflammation and airway microbiota during cystic fibrosis pulmonary exacerbations. Plos One.

[18] Filkins LM (2012). Prevalence of streptococci and increased polymicrobial diversity associated with cystic fibrosis patient stability. J. Bacteriol..

[19] Fodor AA (2012). The adult cystic fibrosis airway microbiota is stable over time and infection type, and highly resilient to antibiotic treatment of exacerbations. Plos One.

[20] Zhao J (2012). Decade-long bacterial community dynamics in cystic fibrosis airways. Proc. Natl. Acad. Sci. USA.

[21] Goffard A (2014). Virus and cystic fibrosis: rhinoviruses are associated with exacerbations in adult patients. J. Clin. Virol. Off. Publ. Pan Am. Soc. Clin. Virol..

[22] Willner D (2012). Case studies of the spatial heterogeneity of DNA viruses in the cystic fibrosis lung. Am. J. Respir. Cell Mol. Biol..

[23] Lysholm F (2012). Characterization of the viral microbiome in patients with severe lower respiratory tract infections, using metagenomic sequencing. Plos One.

[24] Chotirmall SH (2010). Sputum Candida albicans presages FEV_1_ decline and hospital-treated exacerbations in cystic fibrosis. Chest.

[25] Amin R, Dupuis A, Aaron SD, Ratjen F (2010). The effect of chronic infection with Aspergillus fumigatus on lung function and hospitalization in patients with cystic fibrosis. Chest.

[26] Willger SD (2014). Characterization and quantification of the fungal microbiome in serial samples from individuals with cystic fibrosis. Microbiome.

[27] Delhaes L (2018). Prevalence, geographic risk factor, and development of a standardized protocol for fungal isolation in cystic fibrosis: Results from the international prospective study ‘MFIP’. J. Cyst. Fibros..

[28] Armstead J, Morris J, Denning DW (2014). Multi-country estimate of different manifestations of aspergillosis in cystic fibrosis. Plos One.

[29] Middleton PG, Chen SC-A, Meyer W (2013). Fungal infections and treatment in cystic fibrosis. Curr. Opin. Pulm. Med..

[30] Speirs JJ, van der Ent CK, Beekman JM (2012). Effects of Aspergillus fumigatus colonization on lung function in cystic fibrosis. Curr. Opin. Pulm. Med..

[31] Conrad D (2013). Cystic fibrosis therapy: a community ecology perspective. Am. J. Respir. Cell Mol. Biol..

[32] Lim YW (2014). Clinical insights from metagenomic analysis of sputum samples from patients with cystic fibrosis. J. Clin. Microbiol..

[33] Stevens DA (2003). Allergic bronchopulmonary aspergillosis in cystic fibrosis–state of the art: Cystic Fibrosis Foundation Consensus Conference. Clin. Infect. Dis. Off. Publ. Infect. Dis. Soc. Am..

[34] Galand PE, Casamayor EO, Kirchman DL, Lovejoy C (2009). Ecology of the rare microbial biosphere of the Arctic Ocean. Proc. Natl. Acad. Sci. USA.

[35] Sokol H (2017). Fungal microbiota dysbiosis in IBD. Gut.

[36] Kramer R (2015). Cohort Study of Airway Mycobiome in Adult Cystic Fibrosis Patients: Differences in Community Structure between Fungi and Bacteria Reveal Predominance of Transient Fungal Elements. J. Clin. Microbiol..

[37] Delhaes L (2012). The airway microbiota in cystic fibrosis: a complex fungal and bacterial community–implications for therapeutic management. Plos One.

[38] Sanders DB (2010). Failure to recover to baseline pulmonary function after cystic fibrosis pulmonary exacerbation. Am. J. Respir. Crit. Care Med..

[39] Heirali AA (2017). The effects of inhaled aztreonam on the cystic fibrosis lung microbiome. Microbiome.

[40] Bacci G (2016). Pyrosequencing Unveils Cystic Fibrosis Lung Microbiome Differences Associated with a Severe Lung Function Decline. PloS One.

[41] Hogan DA (2016). Analysis of Lung Microbiota in Bronchoalveolar Lavage, Protected Brush and Sputum Samples from Subjects with Mild-To-Moderate Cystic Fibrosis Lung Disease. Plos One.

[42] Cuthbertson L (2016). Respiratory microbiota resistance and resilience to pulmonary exacerbation and subsequent antimicrobial intervention. ISME J..

[43] Bos LDJ, Meinardi S, Blake D, Whiteson K (2016). Bacteria in the airways of patients with cystic fibrosis are genetically capable of producing VOCs in breath. J. Breath Res..

[44] Botterel F (2018). Fungal and Bacterial Diversity of Airway Microbiota in Adults with Cystic Fibrosis: Concordance Between Conventional Methods and Ultra-Deep Sequencing, and Their Practical use in the Clinical Laboratory. Mycopathologia.

[45] Richardson, M., Bowyer, P. & Sabino, R. The human lung and Aspergillus: You are what you breathe in? *Med. Mycol*. **57**, S145–S154 (2019). 10.1093/mmy/myy149 PMC6394755 30816978

[46] Feigelman R (2017). Sputum DNA sequencing in cystic fibrosis: non-invasive access to the lung microbiome and to pathogen details. Microbiome.

[47] Kim SH (2015). Global Analysis of the Fungal Microbiome in Cystic Fibrosis Patients Reveals Loss of Function of the Transcriptional Repressor Nrg1 as a Mechanism of Pathogen Adaptation. Plos Pathog..

[48] Boutin S, Dalpke AH (2017). Acquisition and adaptation of the airway microbiota in the early life of cystic fibrosis patients. Mol. Cell. Pediatr..

[49] Dickson RP (2015). Spatial Variation in the Healthy Human Lung Microbiome and the Adapted Island Model of Lung Biogeography. Ann. Am. Thorac. Soc..

[50] Venkataraman, A. *et al*. Application of a neutral community model to assess structuring of the human lung microbiome. *mBio* **6** (2015). 10.1128/mBio.02284-14 PMC4324308 25604788

[51] Lamoureux Claudie, Guilloux Charles-Antoine, Beauruelle Clémence, Jolivet-Gougeon Anne, Héry-Arnaud Geneviève (2019). Anaerobes in cystic fibrosis patients’ airways. Critical Reviews in Microbiology.

[52] Caverly, L. J. & LiPuma, J. J. Good cop, bad cop: anaerobes in cystic fibrosis airways. *Eur. Respir. J*. **52** (2018). 10.1183/13993003.01146-2018 29997183

[53] Rogers GB, Hoffman LR, Carroll MP, Bruce KD (2013). Interpreting infective microbiota: the importance of an ecological perspective. Trends Microbiol..

[54] Hurley MN, Smyth AR (2018). Staphylococcus aureus in cystic fibrosis: pivotal role or bit part actor?. Curr. Opin. Pulm. Med..

[55] Hector A (2016). Microbial colonization and lung function in adolescents with cystic fibrosis. J. Cyst. Fibros. Off. J. Eur. Cyst. Fibros. Soc..

[56] Zemanick Edith T., Wagner Brandie D., Robertson Charles E., Ahrens Richard C., Chmiel James F., Clancy John P., Gibson Ronald L., Harris William T., Kurland Geoffrey, Laguna Theresa A., McColley Susanna A., McCoy Karen, Retsch-Bogart George, Sobush Kurtis T., Zeitlin Pamela L., Stevens Mark J., Accurso Frank J., Sagel Scott D., Harris J. Kirk (2017). Airway microbiota across age and disease spectrum in cystic fibrosis. European Respiratory Journal.

[57] van Woerden HC (2013). Differences in fungi present in induced sputum samples from asthma patients and non-atopic controls: a community based case control study. BMC Infect. Dis..

[58] Guillot J, Hadina S, Guého E (2008). The genus Malassezia: old facts and new concepts. Parassitologia.

[59] Kerem E (2014). Factors associated with FEV1 decline in cystic fibrosis: analysis of the ECFS patient registry. Eur. Respir. J..

[60] Pages-Monteiro L (2017). Strong incidence of Pseudomonas aeruginosa on bacterial rrs and ITS genetic structures of cystic fibrosis sputa. Plos One.

[61] Whelan FJ (2017). Longitudinal sampling of the lung microbiota in individuals with cystic fibrosis. Plos One.

[62] Somayaji R (2017). Long-term clinical outcomes of ‘Prairie Epidemic Strain’ Pseudomonas aeruginosa infection in adults with cystic fibrosis. Thorax.

[63] Russell GK, Gadhok R, Simmonds NJ (2013). The destructive combination of Scediosporium apiosperum lung disease and exuberant inflammation in cystic fibrosis. Paediatr. Respir. Rev..

[64] Staerck, C. *et al*. The secreted polyketide boydone A is responsible for the anti-Staphylococcus aureus activity of Scedosporium boydii. *FEMS Microbiol. Lett*. **364** (2017). 10.1093/femsle/fnx223 29069388

[65] Vandeputte, P. *et al*. Draft Genome Sequence of the Pathogenic Fungus Scedosporium apiospermum. *Genome Announc*. **2** (2014). 10.1128/genomeA.00988-14 PMC4183877 25278533

[66] Han Z, Kautto L, Nevalainen H (2017). Secretion of Proteases by an Opportunistic Fungal Pathogen Scedosporium aurantiacum. Plos One.

[67] Krüger, W., Vielreicher, S., Kapitan, M., Jacobsen, I. D. & Niemiec, M. J. Fungal-Bacterial Interactions in Health and Disease. *Pathog. Basel Switz*. **8** (2019). 10.3390/pathogens8020070 PMC6630686 31117285

[68] Budden Kurtis F, Shukla Shakti D, Rehman Saima Firdous, Bowerman Kate L, Keely Simon, Hugenholtz Philip, Armstrong-James Darius P H, Adcock Ian M, Chotirmall Sanjay H, Chung Kian Fan, Hansbro Philip M (2019). Functional effects of the microbiota in chronic respiratory disease. The Lancet Respiratory Medicine.

[69] Chiu, L. *et al*. Protective Microbiota: From Localized to Long-Reaching Co-Immunity. *Front. Immunol*. **8** 1678 (2017). 10.3389/fimmu.2017.01678 PMC5725472 29270167

[70] Coron N (2018). Toward the Standardization of Mycological Examination of Sputum Samples in Cystic Fibrosis: Results from a French Multicenter Prospective Study. Mycopathologia.

[71] Abbott J (2009). What defines a pulmonary exacerbation? The perceptions of adults with cystic fibrosis. J. Cyst. Fibros. Off. J. Eur. Cyst. Fibros. Soc..

[72] Paulson JN, Stine OC, Bravo HC, Pop M (2013). Differential abundance analysis for microbial marker-gene surveys. Nat. Methods.

[73] Gevers D (2014). The treatment-naive microbiome in new-onset Crohn’s disease. Cell Host Microbe.

[74] Enaud Raphaël, Hooks Katarzyna B., Barre Aurélien, Barnetche Thomas, Hubert Christophe, Massot Marie, Bazin Thomas, Clouzeau Haude, Bui Stéphanie, Fayon Michael, Berger Patrick, Lehours Philippe, Bébéar Cécile, Nikolski Macha, Lamireau Thierry, Delhaes Laurence, Schaeverbeke Thierry (2019). Intestinal Inflammation in Children with Cystic Fibrosis Is Associated with Crohn’s-Like Microbiota Disturbances. Journal of Clinical Medicine.

[75] Prevaes SMPJ (2016). Development of the Nasopharyngeal Microbiota in Infants with Cystic Fibrosis. Am. J. Respir. Crit. Care Med..

[76] Bernarde C (2015). Impact of the CFTR-potentiator ivacaftor on airway microbiota in cystic fibrosis patients carrying a G551D mutation. Plos One.

[77] Maeda Y (2011). Population structure and characterization of viridans group streptococci (VGS) including Streptococcus pneumoniae isolated from adult patients with cystic fibrosis (CF). J. Cyst. Fibros. Off. J. Eur. Cyst. Fibros. Soc..

[78] Tibshirani R (1997). The lasso method for variable selection in the Cox model. Stat. Med..

[79] Rush, S. T. A. The Phylogenetic LASSO and the Microbiome: Metagenomic Modeling in Fecal Microbiota Transplantation. (2017).

[80] Bach, F. R. Bolasso: model consistent Lasso estimation through the bootstrap. in *Proceedings of the 25th international conference on Machine learning - ICML’08* 33–40 (ACM Press), 10.1145/1390156.1390161 (2008).

