# Supplementary material for: Respiratory mycobiome and suggestion of inter-kingdom network during acute pulmonary exacerbation in cystic fibrosis
Source: Sci Rep. 2020 Feb 27;10:3589. doi: 10.1038/s41598-020-60015-4 (PMC7046743; doi:10.1038/s41598-020-60015-4)
Supplement: Supplementary file 1 — Supplementary Table 2. [file 41598_2020_60015_MOESM1_ESM.docx]

**Title:** Respiratory mycobiome and suggestion of inter-kingdom network during acute pulmonary exacerbation in cystic fibrosis

**Authors:** Perrine Soret^1,2µ^, Louise-Eva Vandenborght^3,4µ^, Florence Francis^5^, Noémie Coron^3,6,7^, Raphael Enaud^3,6,8^, The Mucofong Investigation Group, Marta Avalos^1,2^, Thierry Schaeverbeke^6^, Patrick Berger^3,6^, Michael Fayon^3,6,8^, Rodolphe Thiebaut^1,2,5^, Laurence Delhaes^3,6-9*^

^µ^These two authors contributed equally and are co–first authors.

**Supplementary Table 2.** Types of analysis, codes according to the outcomes, and R packages used in the study.

| Analysis | Exposures | Outcome | | R packages |
| --- | --- | --- | --- | --- |
|  |  | **CFPE clinical status (binary)** | **FEV1 (quantitative)** |  |
| **Network analyses** | Interactions between bacterial and fungal community:  - Permutation-renormalization bootstrap (ReBoot) method |  |  | ccrepe |
| **Bivariate**  **analyses** | Patient’s characteristics at inclusion | - Binary outcome x Quantitative characteristics: T-Student or Wilcoxon-signed-rank test (if T-Student conditions not fulfilled)  - Binary outcome x Qualitative characteristics: Khi-2 test or Fisher exact test (if Khi-2 test conditions not fulfilled) |  | t.test  wilcox.test  chisq.test |
| **Bivariate**  **analyses** | Alpha diversity: Shannon, Simpson, Chao1 | - Binary outcome x Quantitative index: T-Student or Wilcoxon-signed-rank test (if T-Student conditions not fulfilled) | - Quantitative outcome x Quantitative index: test on Pearson correlation coefficient or Spearman's rank correlation coefficient (if Pearson correlation conditions not fulfilled) | vegan  fossil |
| **Bivariate**  **analyses** | Beta diversity: PCoA with Bray-Curtis similarities | Non-parametric Analysis Of SIMilarities (ANOSIM) test |  | vegan  ape |
| **Bivariate**  **Analyses** | - Analysis in whole population: OTU-by-OTU statistical comparisons (genus level)  - Analysis in majority and minority population: OTU-by-OTU statistical comparisons (genus level) | - Model Selection by Testing for the most appropriate distribution: Zero-inflated Gaussian, Zero-inflated negative binomial, Zero-inflated Poisson or non-parametric  - Statistical comparisons are then performed using the most appropriate test: DESeq and EdgeR for discrete distribution, MetagenomeSeq for Zero-inflated Gaussian distribution, Wilcoxon-signed rank-test (in all other cases) |  | wilcox.test |
| **Multivariate**  **Analyses** | - Selection of the most appropriate multivariate method among methods proposed for microbiome data analysis performed by a simulation study (*Soret et al. A simulation framework of high-dimensional phylogenetic microbiota data. In: 29th International Biometric Conference, Jul 2018, Barcelone, Spain*)  - Selected method: Phy-Lasso | Phy-Lasso Logistic regression  - LOO-CV to tune the hyper parameters  - Stability selection of OTU: boostrap procedure | Phy-Lasso Linear regression  - LOO-CV to tune the hyper parameters  - Stability selection of OTU: boostrap procedure | PhyLasso |
